# ModraDoc006, an oral docetaxel formulation in combination with ritonavir (ModraDoc006/r), in metastatic castration‐resistant prostate cancer patients: A phase Ib study

**DOI:** 10.1002/cnr2.1367

**Published:** 2021-03-12

**Authors:** Marit A. C. Vermunt, Debbie G. J. Robbrecht, Lot A. Devriese, Julie M. Janssen, Bas Thijssen, Marianne Keessen, Maarten van Eijk, Rob Kessels, Ferry A. L. M. Eskens, Jos H. Beijnen, Niven Mehra, Andries M. Bergman

**Affiliations:** ^1^ Department of Clinical Pharmacology The Netherlands Cancer Institute Amsterdam The Netherlands; ^2^ Department of Medical Oncology Erasmus MC Cancer Institute Rotterdam The Netherlands; ^3^ Department of Medical Oncology University Medical Center Utrecht Utrecht The Netherlands; ^4^ Department of Pharmacy and Pharmacology The Netherlands Cancer Institute Amsterdam The Netherlands; ^5^ Modra Pharmaceuticals B.V. Amsterdam The Netherlands; ^6^ Department of Biometrics The Netherlands Cancer Institute Amsterdam The Netherlands; ^7^ Department of Pharmaceutical Sciences Utrecht University Utrecht The Netherlands; ^8^ Department of Medical Oncology Radboud University Medical Center Nijmegen The Netherlands; ^9^ Department of Medical Oncology and Oncogenomics The Netherlands Cancer Institute Amsterdam The Netherlands

**Keywords:** chemotherapy, clinical trials, drug discovery and delivery, prostate cancer

## Abstract

**Background:**

ModraDoc006 is an oral formulation of docetaxel, which is co‐administered with the cytochrome P450 3A4 and P‐glycoprotein inhibitor ritonavir (r): ModraDoc006/r. Weekly treatment with ModraDoc006/r had been evaluated in phase I trials in patients with different types of advanced solid tumors, but up to this point in time not in patients with metastatic castration‐resistant prostate cancer (mCRPC).

**Aim:**

We assessed safety and pharmacokinetics (PK) of ModraDoc006/r to establish the recommended phase 2 dose (RP2D) in patients with mCRPC.

**Methods:**

mCRPC patients, treatment naïve or following abiraterone or enzalutamide treatment, were included. Dose‐escalation of ModraDoc006/r was based on safety and docetaxel PK. Antitumor activity was assessed by serum prostate‐specific antigen (PSA) and radiological evaluation.

**Results:**

Cohort 1 (*n* = 5) received once weekly ModraDoc006 30 mg with ritonavir 100 mg in the morning, and ModraDoc006 20 mg with ritonavir 100 mg in the evening (30‐20/100‐100). The mean docetaxel area under the plasma concentration‐time curve (mAUC0‐inf) was 461 ng/mL × h with 1 dose limiting toxicity (DLT); grade 3 alanine transferase increase. In cohort 2 (*n* = 6, ModraDoc006/r 30‐20/200‐200), the mAUC0‐inf was 1687 ng/mL × h with 2 DLTs; grade 3 diarrhea and mucositis. In cohort 3A (*n* = 6, ModraDoc006/r 30‐20/200‐100), the mAUC0‐inf was 1517 ng/mL × h with 1 DLT; grade 3 diarrhea. In cohort 3B (*n* = 3, ModraDoc006/r 20‐20/200‐100), the mAUC0‐inf was 558 ng/mL × h without DLTs. The mAUC0‐inf exceeded estimated exposures of intravenous docetaxel in cohort 2 and 3A, was lower in cohort 1 and was in range in cohort 3B. PSA decreases of >50% occurred in 6/10 evaluable patients throughout the various cohorts. In five radiological evaluable patients, two confirmed partial responses were observed.

**Conclusion:**

The RP2D was established at weekly ModraDoc006/r 30‐20/200‐100. Observed PSA and radiological responses suggest promising clinical activity. These results have led to an ongoing randomized Phase 2b study, comparing weekly ModraDoc006/r with 3‐weekly IV docetaxel in patients with mCRPC.

## 1 INTRODUCTION

In 2004, docetaxel was the first drug to establish an overall survival benefit in patients with metastatic castration‐resistant prostate cancer (mCRPC) and has been standard of care since.^1^ Intravenous (IV) docetaxel is commonly dosed in a 3‐weekly schedule, with neutropenia and neuropathy being dose limiting. Weekly docetaxel is comparably active with less myelosuppression.^1^, ^2^ Unfortunately, weekly infusions are inconvenient for patients and are seldom used in practice.

Oral administration of anticancer drugs is often preferred by patients over IV administration.^3^ Moreover, docetaxel formulated as an oral drug does not require dexamethasone prophylaxis and can be more cost‐effective.^4^, ^5^ However, oral administration is difficult due to a low bioavailability of docetaxel after oral intake. Oral docetaxel bioavailability is pharmacologically hampered by the drug efflux pump P‐glycoprotein (P‐gp) and the eliminating enzyme cytochrome P450 (CYP)3A4—both effects have been addressed by co‐administration of ritonavir, which inhibits both CYP3A4 and P‐gp.^6^, ^7^, ^8^ In addition, it is suggested that ritonavir may increase the antitumor activity of docetaxel by inhibition of the CYP3A4‐mediated metabolism of the drug within prostate cancer cells.^9^ Furthermore, the oral bioavailability of docetaxel is pharmaceutically hampered by its low water solubility, which was solved by production of a solid docetaxel dispersion containing a hydrophilic carrier and surfactant, known in tablet form as ModraDoc006.^10^

ModraDoc006 with ritonavir (ModraDoc006/r) treatment has been investigated in patients with different solid tumors, but not specifically in mCRPC patients.^11^ A recent meta‐analysis revealed that mCRPC patients treated with IV docetaxel have a lower docetaxel exposure compared to patients with other solid tumors.^12^ This warrants optimal dose assessment of ModraDoc006/r, in particular in patients with mCRPC. We therefore explored the safety, pharmacokinetics (PK) and recommended phase 2 dose (RP2D) of weekly ModraDoc006/r in patients with mCRPC, as first treatment or following abiraterone or enzalutamide.

## 2 METHODS

### 2.1 Study design and treatment

The primary aim of this multicenter open label phase Ib study was to establish the RP2D of ModraDoc006/r in a once weekly bi‐daily (BID) schedule in mCRPC patients. Dose‐escalation and establishment of the RP2D were based on the maximum tolerated dose (MTD) and PK results. Cohort 1 was dosed at the RP2D of the previous phase 1 study; 30 mg ModraDoc006 plus 100 mg ritonavir in the morning, followed by 20 mg ModraDoc006 plus 100 mg ritonavir in the evening (30‐20/100‐100).^11^

During the first two weekly cycles, patients were admitted to the hospital for supervised administration of ModraDoc006/r (docetaxel as ModraDoc006 10 mg tablets and ritonavir as Norvir 100 mg tablets) with 7 hours between the morning and evening dose, while fasting 1 hour before and after the administration. Granisetron premedication was given before every administration during the first two cycles and upon indication in subsequent cycles. Daily prednisolone (BID 5 mg) was started with the study treatment. Patients received no dexamethasone premedication. From cycle 3 onwards, ModraDoc006/r was used at home in a once weekly schedule (with 7‐10 hours between the two daily administrations) for a maximum of 30 cycles. Early drug discontinuation was pursued in case of progressive disease (PD), inadequate docetaxel exposure or grade ≥ 3 related adverse events (AEs) despite a maximum of two dose reductions.

### 2.2 Patient eligibility

Patients with mCRPC considered eligible for standard palliative docetaxel were enrolled. All patients were treatment naïve or had previously received abiraterone or enzalutamide for castration‐resistant disease. A World Health Organization Performance Status (WHO PS) of ≤2 and life expectancy of ≥3 months were required. Castration‐resistant disease was defined as biochemical and/or radiological progression according to the Prostate Cancer Working Group 3 (PCWG3) recommendations.^13^ Hemoglobin levels of ≥10 g/dL, absolute neutrophil counts of ≥1.5 × 10^9^/L, platelet counts of ≥100 × 10^9^/L, serum testosterone levels of ≤50 ng/dL (≤1.73 nmol/L) and adequate hepatic and renal functions were required for inclusion. Patients with bowel obstructions or motility disorders that could hamper the intake or absorption of drugs were excluded. The use of concomitant CYP3A4 or P‐gp modulating drugs was not allowed, including bicalutamide <14 days prior to start of ModraDoc006/r. Prior treatment with chemotherapy was not allowed.

The study was performed in accordance with current standards of International Conference for Harmonization Good Clinical Practice, the WHO Declaration of Helsinki and the Medical Research Involving Human Subjects Act. The study was approved by the medical ethical boards of all participating hospitals and registered (European Union Drug Regulating Authorities Clinical Trials Database 2016‐005056‐13 and clinicaltrials.gov ID NCT03136640).

### 2.3 Safety

Weekly safety assessments during the first 10 weeks and every subsequent 2 weeks included routine physical examination, vital signs, WHO PS, hematology and chemistry tests and recording of AEs and concomitant medication. AEs were evaluated according to the Common Terminology Criteria for AEs (CTCAE) version 4.03. Dose limiting toxicities (DLTs) were defined as grade ≥ 3 AEs that were possibly, probably, or definitely related to ModraDoc006/r, occurring in the first 4 weeks of treatment despite optimal supportive care. The following toxicities were considered DLTs: grade ≥ 3 nonhematologic toxicity, grade ≥ 4 anemia and thrombocytopenia or grade 3 thrombocytopenia with bleeding, grade ≥ 3 (febrile) neutropenia or inability to continue study treatment within 7 days of scheduled dosing due to toxicity related to ModraDoc006/r. If ≥2/6 patients experienced a DLT, this dose was considered nontolerable. For the MTD, ≤1 dose limiting toxicity (DLT) in six patients was allowed.

Patients who received ≥1 dose of ModraDoc006/r were evaluable for AEs. Patients who did not complete the first four treatment cycles due nondrug‐related events or had clinically relevant drug‐drug interactions (DDIs), were considered nonevaluable for DLTs and were replaced. Safety follow‐up continued until 28 days after the last intake of ModraDoc006/r.

### 2.4 Pharmacokinetics

Venous blood samples for PK analysis were obtained at 16 time‐points up to 48 hours after intake of the first two cycles of ModraDoc006/r. Because of the influence of the CYP3A4‐inducer enzalutamide on the PK of ModraDoc006/r in patients in cohort 1, sampling in the subsequent cohorts was performed at cycle 1 and cycle ≥5 in patients using enzalutamide ≤28 days prior to the first administration of ModraDoc006/r.^14^ Blood samples were collected in 4 mL lithium heparin tubes, centrifuged at 1500*g* at 4°C for 10 minutes and stored at −20°C within 1 hour after sampling. Plasma docetaxel and ritonavir concentrations were measured by a validated bioanalytical assay with a lower limit of quantification of docetaxel and ritonavir of 0.5 and 2.0 ng/mL, respectively.^15^ Docetaxel and ritonavir PK characteristics were quantified by noncompartmental analyses, using the R software (version 3.6.1).^16^ For all cohorts, to ensure adequate PK and in view of potential intra‐patient variation, a relatively high pre‐specified target (mAUC_0‐inf_ of 800 ng/mL × h) was applied in our study to guide our dose‐escalation, based on the reported estimated weekly exposure of 600 ng/mL × h with IV docetaxel in mCRPC patients.^12^

### 2.5 Antitumor activity

Patients that received ≥9 weekly cycles of ModraDoc006/r were considered evaluable for response. This was assessed with prostate‐specific antigen (PSA) measurements, computed tomography and bone scintigraphy every 6 weeks. Biochemical response was defined as a PSA decline of ≥50% from baseline (≤2 weeks before start), preferably confirmed by a second PSA value ≥4 weeks later.^13^ Radiological response was defined as a complete or partial regression of measurable target lesions (RECIST version 1.1) compared to baseline, confirmed by a second scan ≥6 weeks later. Radiographic progression was assessed per RECIST criteria and bone progression according to the PCWG3 recommendations.^13^

## 3 RESULTS

### 3.1 Patient characteristics and study treatment

Baseline characteristics of the patients are summarized in Table 1. Of the 24 enrolled patients, 1 did not start with ModraDoc006/r and 2 were included in the baseline population but not in the PK and safety analysis because of DDIs. All patients had bone metastases, while 43% also had lymph node metastases and 35% had visceral metastases. Thirteen out of 23 (57%) patients had received prior therapy with either enzalutamide or abiraterone. In three out of eight patients with prior enzalutamide, this was discontinued <28 days prior to initiation of ModraDoc006/r. In total, five patients had a DDI with ModraDoc006/r (one with mirabegron, one with methotrexate, three with enzalutamide). At study enrollment, 4 patients had a biochemical PD only, 8 patients had radiographic progression only, while 11 patients had combined PD.

**TABLE 1:** Patient baseline characteristics

Characteristics	Cohort 1 30‐20/100‐100 (*N* = 5)	Cohort 2 30‐20/200‐200 (*N* = 8)^a^	Cohort 3A 30‐20/200‐100 (*N* = 7)^b^	Cohort 3B 20‐20/200‐100 (*N* = 3)^c^
Age				
Mean (range), years	64 (54‐68)	73 (63‐82)	70 (60‐76)	64 (55‐76)
BSA				
Mean (range), m^2^	2.23 (2.13‐2.36)	2.10 (1.83‐2.59)	1.97 (1.71‐2.41)	2.06 (1.81‐2.35)
Ethnicity				
Caucasian African descent (black)	5 (100%) —	8 (100%) —	6 (86%) 1 (14%)	3 (100%) —
WHO PS				
0 1 2	5 (100%) — —	2 (25%) 5 (63%) 1 (13%)	3 (43%) 4 (57%) —	1 (33%) 2 (67%) —
Chronic comorbidities				
Cardiovascular^d^ Pulmonary^e^ Diabetes mellitus type 2 Immune diseases^f^	3 (60%) 2 (40%) 1 (20%) 1 (20%)	7 (88%) 2 (25%) 2 (25%) 1 (13%)	4 (57%) — 2 (29%) 2 (29%)	1 (33%) 1 (33%) 2 (67%) 1 (33%)
Sites of metastasis				
Lymph nodes Bone Visceral^g^	3 (60%) 5 (100%) —	3 (38%) 8 (100%) 3 (38%)	2 (29%) 7 (100%) 3 (43%)	2 (67%) 3 (100%) 2 (67%)
Prior therapy for mCRPC				
Enzalutamide <28 days prior to start Abiraterone Chemotherapy Samarium Radium‐223	3 (60%) 2 — — 1 (20%)	2 (25%) 1 1 (13%) — — —	3 (43%) — 3 (43%) — — 1 (14%)	— — 1 (33%) — 1 (33%) —
Type of progression				
PSA only Soft tissue only Bone only PSA and bone PSA and soft tissue PSA, soft tissue, bone	— 1 (20%) — 2 (40%) 1 (20%) 1 (20%)	1 (13%) 1 (13%) 3 (38%) 2 (25%) 1 (13%) —	1 (14%) — 2 (29%) 3 (43%) — 1 (14%)	2 (67%) — 1 (33%) — — —
Baseline PSA				
Median (range), μg/L	134.70 (21.02‐776.60)	41.50 (2.10‐2100.00)	51.00 (0.10‐180.00)	7.39 (1.70‐26.00)

Abbreviations: BSA, body surface area; mCRPC, metastatic castration‐resistant prostate cancer; *N*, number of patients; PSA, prostate‐specific antigen; WHO PS, World Health Organization performance status.

^a^ Eight patients were included, of whom two patients were considered nonevaluable because of a drug‐drug interaction due to use of mirabegron or discontinuation after only treatment cycle due to development of a sepsis unrelated to the study treatment.

^b^ Seven patients were included, of whom one patient was considered nonevaluable because of concomitant use of low dose methotrexate.

^c^ Four patients were enrolled, of whom one patient did not start in the study due to rapid clinical deterioration. This patient is not included in the safety population.

^d^ Pulmonary conditions including: asthmatic/chronic obstructive pulmonary disease and sarcoidosis.

^e^ Cardiovascular conditions including: hypertension, aortic aneurysm, myocardial infarction/angina pectoris, venous thrombotic events with chronic anticoagulant use, cardiac rhythm disorders.

^f^ Immune diseases including: psoriasis, immune hepatitis, lichen planus, polymyalgia rheumatica, hypothyroidism.

^g^ Visceral metastasis including lesions in: liver, lung, bladder, adrenal gland.

**FIGURE 1:**
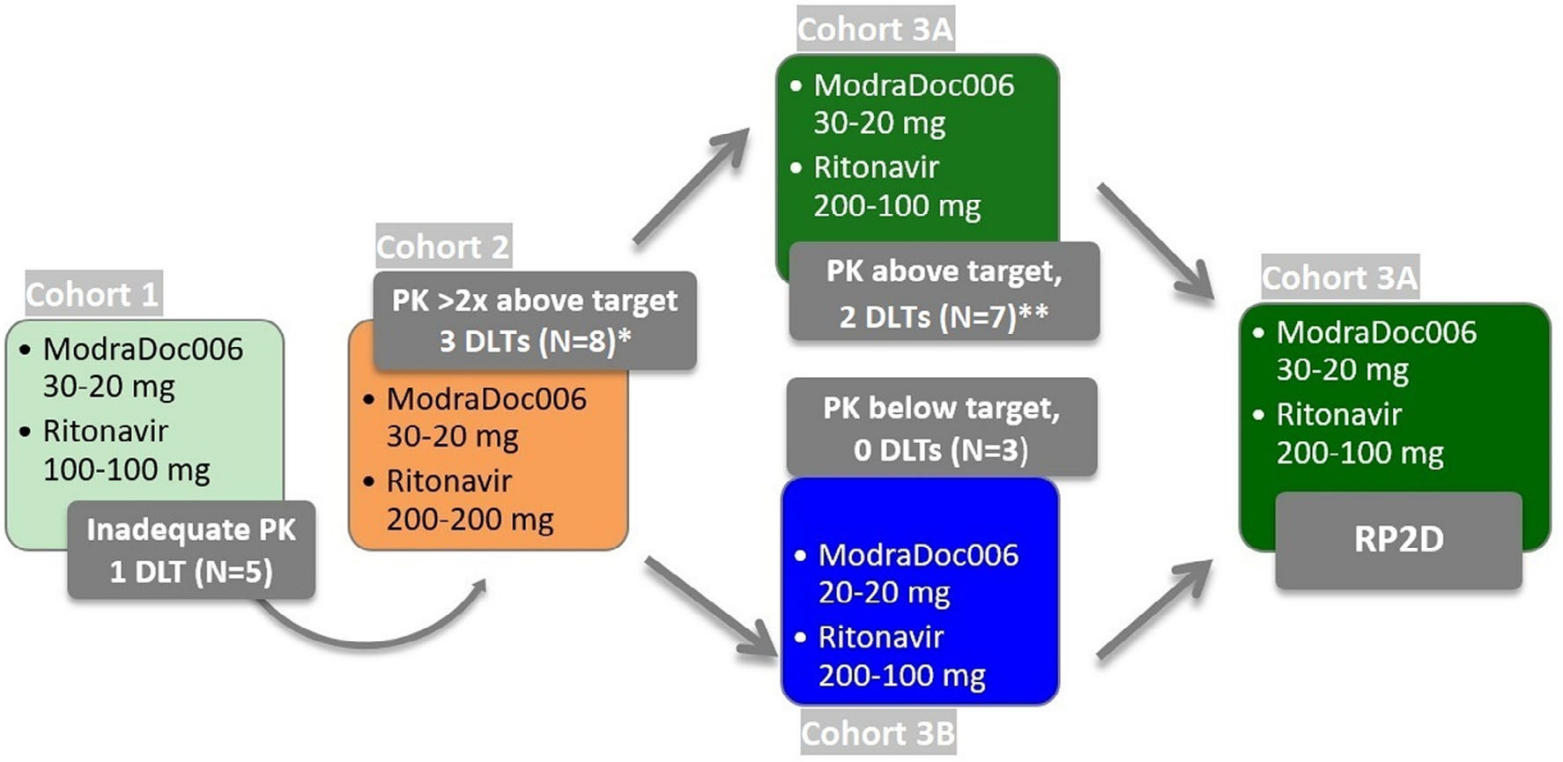
Dose levels and evaluations. Dosing and results of once weekly ModraDoc006/r per cohort. In cohort 1, patients were treated on the RP2D established in the earlier phase 1 trial in patients with different types of solid tumors.^11^ *One patient with a DLT was considered nonevaluable because of concomitant use of the P‐gp inhibitor mirabegron and **one patient with a DLT was considered nonevaluable because of the concomitant use of low dose oral methotrexate. DLT, dose limiting toxicity; *N*, number of patients; PK, pharmacokinetic exposure; RP2D, recommended phase 2 dose

As described in Figure 1, cohort 1 was treated with ModraDoc006/r 30‐20/100‐100 (*n* = 5) and cohort 2 with 30‐20/200‐200 (*n* = 8). In the simultaneously evaluated cohorts 3A and 3B, patients were treated with 30‐20/200‐100 (*N* = 7) and 20‐20/200‐100 (*n* = 3), respectively.

**FIGURE 2:**
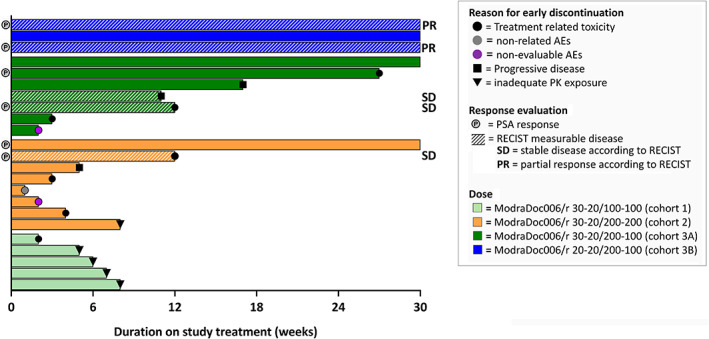
Duration on study and evaluations per patient. Duration of treatment with ModraDoc006/r in weeks, the reason for early discontinuation if indicated and treatment response. Each bar represents one individual patient. Patients with PSA responses are depicted on the left. The striped bars indicate that the patient has measurable disease according to RECIST, the corresponding evaluation (SD or PR) is depicted at the end of the bar on the right. Each dose level is represented by one color, as stated in the legend

Figure 2 summarizes the treatment duration and evaluations per patient. Five out of 23 (22%) patients completed all 30 ModraDoc006/r cycles. The remaining 18 (78%) patients discontinued before 30 cycles because of drug‐related AEs (7 patients), disease progression (3 patients), switch to standard of care based on the PK results (4 patients in cohort 1 and 1 patient in cohort 2), toxicity related to a DDI with ModraDoc006/r (2 patients) and nondrug‐related AEs (1 patient).

### 3.2 Safety

All drug‐related AEs of CTCAE grade ≥ 2 are summarized in Table 2, including the DLTs. Grade ≥ 2 drug‐related toxicities, occurring in >10% of patients, were fatigue, anorexia, diarrhea, nausea, dyspepsia and nail toxicity. Except in the two patients with a DDI due to mirabegron and methotrexate, no febrile neutropenia was observed.

**TABLE 2:** Treatment related adverse events

Adverse events^a^	Gr	Cohort 1 30‐20/100‐100 (*N* = 5)	Cohort 2 30‐20/200‐200 (*N* = 8)	Cohort 3A 30‐20/200‐100 (*N* = 7)	Cohort 3B 20‐20/200‐100 (*N* = 3)	All cohorts (*N* = 23)
Hematological						
Febrile neutropenia	3	—	13% (1)^b^	14% (1)^c^	—	9% (2)^b^ ^,^ ^c^
Leucopenia	3	—	13% (1)^b^	—	—	4% (1)^b^
Anemia	2 3	— —	— —	— 14% (1)^c^	33% (1) 33% (1)	4% (1) 9% (2)^c^
Gastro‐intestinal						
**Diarrhea**	2 3	— —	13% (1) 25% (2)	29% (2)^c^ 29% (2)	33% (1) —	17% (4)^c^ 17% (4)
**Oral mucositis**	2 3	— —	— 13% (1)	14% (1)^c^ —	— —	4% (1) 4% (1)
**Gastro‐intestinal mucositis**	3	—	13% (1)	—	—	4% (1)
Nausea	2	40% (2)	13% (1)^b^	14% (1)	—	17% (4)
Vomiting	2	20% (1)	—	14% (1)	—	9% (2)
Anorexia	2 3	— —	38% (3) —	14% (1) 14% (1)	33% (1) —	22% (5) 4% (1)
Dysgeusia	2	—	—	14% (1)	—	4% (1)
Dyspepsia	2	—	13% (1)	29% (2)	—	13% (3)
Abdominal pain	2	—	—	29% (2)^c^	—	9% (2)^c^
Constipation	2	—	13% (1)	—	33% (1)	9% (2)
Other						
Fatigue	2 3	— —	38% (3) 13% (1)^b^	43% (3) —	33% (1) —	30% (7) 4% (1)^b^
**ALAT increase**	2 3	— 20% (1)	— —	14% (1)^c^ —	— —	4% (1)^c^ 4% (1)
ASAT increase	2	—	—	14% (1)^c^	—	4% (1)^c^
Hypotension	2	—	25% (2)	—	—	9% (2)
Malaise	2	—	13% (1)^b^	—	—	4% (1)^b^
Weight loss	2	—	—	14% (1)	—	4% (1)
Rhinitis	2	—	—	14% (1)	—	4% (1)
Balanitis	2	—	—	14% (1)	—	4% (1)
Peripheral neuropathy	2	—	—	14% (1)	—	4% (1)
Ascites	2	—	13% (1)	—	—	4% (1)
Alopecia	2	—	13% (1)	—	—	4% (1)
Nail toxicity	2	—	13% (1)	29% (2)	—	13% (3)
Paronychia	2	—	—	14% (1)	—	4% (1)
Dry skin	2	—	13% (1)	—	—	4% (1)
Skin fissures	2	—	13% (1)	—	—	4% (1)

*Note*: In case of multiple grades of the same AE, the worst grade was reported per patient. DLTs are highlighted in bold.

Abbreviations: ALAT, serum alanine aminotransferase; ASAT, serum aspartate aminotransferase; *N*, number of patients.

^a^ All CTCAE grade (Gr) ≥ 2 adverse events (AEs) that were possibly, probably or definitely related to ModraDoc006/r.

^b^ Including the nonevaluable patient using mirabegron.

^c^ Including the nonevaluable patient using methotrexate.

As described in Figure 1, 1 DLT (grade 3 serum alanine transferase increase) was observed in cohort 1 (*n* = 5). In cohort 2 (*n* = 6), 2 patients experienced DLTs (grade 3 diarrhea and mucositis). Another patient in this cohort experienced grade 3 febrile neutropenia, mucositis and fatigue, which was considered nonevaluable toxicity due to a DDI with mirabegron. In cohort 3A (*n* = 6), 1 DLT was observed (grade 3 diarrhea). Another patient experienced grade 3 febrile neutropenia and grade 2 mucositis, which was considered nonevaluable toxicity due to a DDI with oral methotrexate. In cohort 3B (*n* = 3), no DLTs were observed. Therefore, based on the safety and observation of 1 DLT in six evaluable patients, the MTD was established as 30‐20/200‐100 (Cohort 3A).

### 3.3 Pharmacokinetics

**FIGURE 3:**
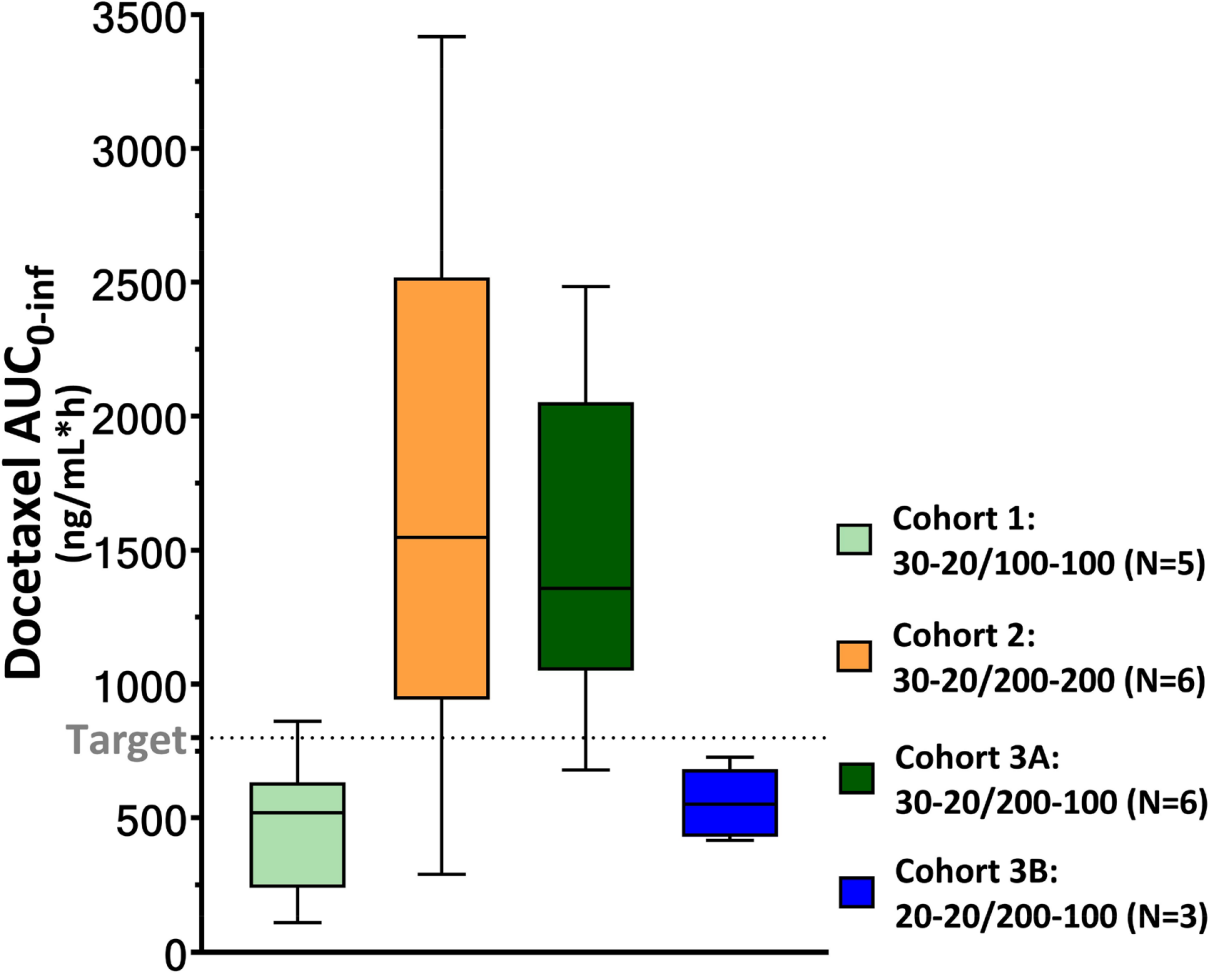
Docetaxel exposure per cohort. Weekly docetaxel area under the plasma concentration vs time curve from zero to infinity (AUC_0‐inf_) per cohort. For each patient, the AUC_0‐inf_ of two treatment cycles of ModraDoc006/r was included. Each dose level is represented by one color, as stated in the legend. Each box represents the median AUC_0‐inf_ with the 25% and 75% percentile and the whiskers indicate the minimum and maximum AUC_0‐inf_ that was observed in the cohort. The pre‐specified exposure target is indicated by the dotted line

Detailed PK results are included in Table S1 and the individual plasma concentration vs time curves of the patients in the last two cohorts are provided in Figure S1A,B. The docetaxel AUC_0‐inf_ per cohort is depicted in Figure 3.

In cohort 1 (*n* = 5, ModraDoc006/r 30‐20/100‐100), in cycle 1, the docetaxel mAUC_0‐inf_ was 399 ng/mL × h (coefficient of variation [CV] 49.6%) with a mean maximum plasma concentration (mC_max_) of 33.0 ng/mL (CV 55.5%). In cycle 2, the mAUC_0‐inf_ was 524 ng/mL × h (CV 55.5%) and mC_max_ was 46.1 ng/mL (CV 69.3%).

In cohort 2 (*n* = 6) with a doubling of the ritonavir dose (ModraDoc006/r 30‐20/200‐200), in cycle 1, the docetaxel mAUC_0‐inf_ (1554 ng/mL × h (CV 67.9%) and mC_max_ (146 ng/mL, CV 56.3%) increased. In cycle 2, the mAUC_0‐inf_ was 1821 ng/mL × h (CV 56.5%) and mC_max_ was 150.3 ng/mL (CV 46.5%).

In cohort 3A (*n* = 6, ModraDoc006/r 30‐20/200‐100), in cycle 1, the docetaxel mAUC_0‐inf_ was 1342 ng/mL × h (CV 39.6%) with a mC_max_ of 164 ng/mL (CV 30.2%). In cycle 2, the mAUC_0‐inf_ and mC_max_ were 1692 ng/mL × h (CV 40.0%) and 170 ng/mL (CV 40.2%).

In cohort 3B (*n* = 3, ModraDoc006/r 20‐20/200‐100), in cycle 1, the mAUC_0‐inf_ was 526 (CV 33.2%) and in cycle 2 this was 590 ng/mL × h (CV 12.1%), with corresponding mC_max_ values of 46.1 (CV 46.8%) and 51.9 ng/mL (CV 45.7%).

To investigate DDIs, additional PK sampling was performed in a patient in cohort 1, who used enzalutamide up to 9 days before initiation of ModraDoc006/r. This might have lowered his docetaxel AUC_0‐inf_ at cycle 1 and 2 (485 and 610 ng/mL × h) as it increased (934 ng/mL × h) at cycle 8. Another patient in cohort 2 had an extremely high docetaxel AUC_0‐inf_ (3363 and 4106 ng/mL × h at cycle 1 and 2), potentially due to a DDI with the P‐gp inhibitor mirabegron.

## 4 RP2D

Based on both the MTD and the PK, the RP2D of ModraDoc006/r was established as 30‐20/200‐100 (Cohort 3A). This dose resulted in 1 DLT in six patients and a docetaxel mAUC_0‐inf_ of 1517 ng/mL × h (CV 40.1%), which exceeds the pre‐specified exposure target of 800 ng/mL × h.

### 4.1 Antitumor activity

As summarized in Figure 2, PSA responses (≥50% decline) were observed in 6 out of 10 evaluable patients (treated with ≥9 ModraDoc006/r cycles). In five patients with RECIST measurable disease, two confirmed partial responses (both in cohort 3B) were observed.

## 5 DISCUSSION

In this study, we assessed the safety and PK of weekly ModraDoc006/r and established the RP2D dose as 30‐20/200‐100 in mCRPC patients. This RP2D differs from the dose in patients with other solid tumors, based on differences in the PK profile of ModraDoc006/r.^11^ These results are in line with reported differences for IV treatment with docetaxel, where a higher clearance was observed in mCRPC patients as compared to patients with other solid tumors.^12^ It is suggested that this can be attributed to effects of medical castration.^17^ Despite a higher docetaxel clearance, a similar CYP3A4 activity was observed in castrated vs noncastrated prostate cancer patients.^17^ As the expression of drug transporters and hepatic uptake of docetaxel was increased in castrated animal models, it is hypothesized that this results in a higher CYP3A4‐mediated clearance.^17^ In addition, with oral docetaxel treatment, inhibition of CYP3A4 by ritonavir is required for the systemic uptake. Because ritonavir is a CYP3A4 substrate itself, a higher clearance by CYP3A4 results in lower plasma concentrations of this booster drug, as was indeed observed in this study. The combination of a higher clearance and lower uptake will lead to a lower docetaxel exposure in mCRPC patients treated with ModraDoc006/r. This problem was successfully addressed by increasing the ritonavir dose in the subsequent cohorts.

The reported mAUC_0‐inf_ of 3‐weekly IV docetaxel (75 mg/m^2^) in mCRPC is approximately 1820 ng/mL × h.^12^ Extrapolating to a weekly schedule, an equivalent docetaxel AUC_0‐inf_ would be ± 600 ng/mL × h. When extrapolated from IV docetaxel, cohort 1 (30‐20/100‐100) resulted in mAUCs below what would be expected with IV docetaxel, the mAUC_0‐inf_ in cohorts 2 and 3A (30‐20/200‐200 and 30‐20/200‐100, respectively) was substantially higher and cohort 3B (20‐20/200‐100) was in range of IV docetaxel. Based on the high docetaxel exposure and acceptable safety with 1 DLT in 6 evaluable patients, cohort 3A was considered as the MTD, which was the primary endpoint of this trial. However, although the docetaxel exposure was lower, in cohort 3B, all three patients completed the maximum of 30 weekly cycles of ModraDoc006/r with good tolerance and promising signs of activity. Considering ModraDoc006/r's potential role as a more tolerable treatment alternative to IV docetaxel in a palliative setting, ModraDoc006/r 20‐20/200‐100 may also be considered as a promising dose level that could be further explored. In that context, we decided to pursue these investigations in a subsequent randomized phase 2b trial. This trial is currently ongoing, with a direct comparison of both ModraDoc006/r 30‐20/200‐100 (cohort 3A) and 20‐20/200‐100 (cohort 3B) to standard IV docetaxel in mCRPC patients (clinicaltrials.gov ID NCT04028388). Additional pharmacokinetic data for the ModraDoc006/r 20‐20/200‐100 dose will be obtained in an ongoing phase I trial in earlier stage prostate cancer patients (clinicaltrials.gov ID NCT03066154).

Given this phase I trial was relatively small and conducted in four Dutch hospitals, we were not able to enroll a racially diverse patient population. As is known from standard IV treatment, the pharmacokinetics, toxicity and possibly the efficacy of treatment with docetaxel can be influenced by ethnicity.^18^, ^19^ The larger ongoing follow‐up trial, conducted in multiple countries including the United States, will hopefully enroll more patients of noncaucasian descent.

Although direct comparison has to be awaited, the hematological toxicity of ModraDoc006/r seems lower in comparison to IV docetaxel, which is consistent with prior clinical studies; grade 3‐4 neutropenia was not observed in this study, except in the two patients with a DDI, compared with at least 30% of the mCRPC patients treated with the 3‐weekly IV schedule.^1^, ^2^, ^11^ Several studies showed that neutropenia is correlated to the PK, especially to the AUC of docetaxel.^20^, ^21^ Although the reported relationship between C_max_ and neutropenia is not definitively established, the lower neutropenia rate with ModraDoc006/r may be explained by an up to 10‐fold lower C_max_ of docetaxel with ModraDoc006/r treatment as compared to IV docetaxel.^22^ In contrast, diarrhea and mucositis are the major DLTs of weekly ModraDoc006/r.

## 6 CONCLUSION

Based upon the safety and PK evaluation, we defined the RP2D of ModraDoc006/r as 30‐20/200‐100. Although the docetaxel exposure was lower, though in range with IV docetaxel, ModraDoc006/r 20‐20/200‐100 may also be considered for further exploration. The observed safety, PK profile and preliminary antitumor activity in both dose levels underline, in our view, ModraDoc006/r as a safe and convenient alternative to IV docetaxel. These results have led to an ongoing randomized Phase 2b study, comparing weekly ModraDoc006/r with 3‐weekly IV docetaxel in patients with mCRPC.

## AUTHOR CONTRIBUTIONS

**Marit Vermunt:** Conceptualization; formal analysis; investigation; methodology; project administration; writing‐original draft. **Debbie Robbrecht:** Formal analysis; investigation; project administration; writing‐review & editing. **Lot Devriese:** Formal analysis; investigation; project administration; writing‐review & editing. **Julie Janssen:** Formal analysis; writing‐review & editing. **Bas Thijssen:** Formal analysis; writing‐review & editing. **Marianne Keessen:** Project administration; writing‐review & editing. **Maarten van Eijk:** Formal analysis; writing‐review & editing. **Rob Kessels:** Data curation; formal analysis; writing‐review & editing. **Ferry Eskens:** Formal analysis; investigation; project administration; writing‐review & editing. **Jos Beijnen:** Conceptualization; funding acquisition; writing‐review & editing. **Niven Mehra:** Formal analysis; investigation; project administration; writing‐review & editing. **Andre Bergman:** Conceptualization; formal analysis; investigation; methodology; project administration; supervision; writing‐review & editing.

## CONFLICT OF INTEREST

Modra Pharmaceuticals B.V. was founded as a spin‐off company of the Netherlands Cancer Institute, enabling the further clinical development of novel oral taxane formulations, after their pharmaceutical and preclinical development in the Netherlands Cancer Institute. Jos H. Beijnen is a (part‐time) employee and shareholder of Modra Pharmaceuticals B.V. and is patent holder on oral taxane formulations. Marianne Keessen is a full‐time employee of Modra Pharmaceuticals B.V. The other authors declare no conflicts of interest.

## ETHICAL STATEMENT

The study was approved by medical ethical board of all participating hospitals. The study was performed in accordance with the Declaration of Helsinki. All patients signed informed consent before the start of any study‐related procedures.

## Supporting information

**Data S1**. Supporting Information.

## Data Availability

The data that support the findings of this study are available on request from the corresponding author. The data are not publicly available due to privacy and ethical restrictions.

## References

[1] Tannock IA, de Wit R, Berry WR, et al. Docetaxel plus prednisone or mitoxantrone plus prednisone for advanced prostate cancer. N Engl J Med. 2004;351(15):1502‐1512. 15470213 10.1056/NEJMoa040720

[2] Engels FK, Verweij J. Docetaxel administration schedule: from fever to tears? A review of randomised studies. Eur J Cancer. 2005;41(8):1117‐1126. 15911234 10.1016/j.ejca.2005.02.016

[3] Liu G, Franssen E, Fitch MI, Warner E. Patient preferences for oral versus intravenous palliative chemotherapy. J Clin Oncol. 1997;15(1):110‐115. 8996131 10.1200/JCO.1997.15.1.110

[4] Markman M. Managing taxane toxicities. Support Care Cancer. 2003;11(3):144‐147. 12618923 10.1007/s00520-002-0405-9

[5] Armstrong A, Bui C, Fitch K, et al. Docetaxel chemotherapy in metastatic castration‐resistant prostate cancer: cost of care in medicare and commercial populations. Cur Med Res Opin. 2017;33(6):1133‐1139. 10.1080/03007995.2017.1308919 28318331

[6] van Waterschoot RA, Lagas JS, Wagenaar E, et al. Absence of both cytochrome P450 3A and P‐glycoprotein dramatically increases docetaxel oral bioavailability and risk of intestinal toxicity. Cancer Res. 2009;69(23):8996‐9002. 19920203 10.1158/0008-5472.CAN-09-2915

[7] Bardelmeijer HA, Ouwehand M, Buckle T, et al. Low systemic exposure of oral docetaxel in mice resulting from extensive first‐pass metabolism is boosted by ritonavir. Cancer Res. 2002;62(21):6158‐6164. 12414642

[8] Oostendorp RL, Huitema A, Rosing H, et al. Coadministration of ritonavir strongly enhances the apparent oral bioavailability of docetaxel in patients with solid tumors. Clin Cancer Res. 2009;15(12):4228‐4233. 19509162 10.1158/1078-0432.CCR-08-2944

[9] Ikezoe T, Hisatake Y, Takeuchi T, et al. HIV‐1 protease inhibitor, ritonavir: a potent inhibitor of CYP3A4, enhanced the anticancer effects of docetaxel in androgen‐independent prostate cancer cells in vitro and in vivo. Cancer Res. 2004;64(20):7426‐7431. 15492266 10.1158/0008-5472.CAN-03-2677

[10] Sawicki E, Beijnen JH, Schellens JHM, Nuijen B. Pharmaceutical development of an oral tablet formulation containing a spray dried amorphous solid dispersion of docetaxel or paclitaxel. Int J Pharm. 2016;511(2):765‐773. 27480397 10.1016/j.ijpharm.2016.07.068

[11] De Weger VA, Stuurman FE, Hendrikx JJMA, et al. A dose‐escalation study of bi‐daily once weekly oral docetaxel either as ModraDoc001 or ModraDoc006 combined with ritonavir. Eur J Cancer. 2017;86:217‐225. 29031170 10.1016/j.ejca.2017.09.010

[12] de Vries Schultink AHM, Crombag MBS, van Werkhoven E, et al. Neutropenia and docetaxel exposure in metastatic castration‐resistant prostate cancer patients: a meta‐analysis and evaluation of a clinical cohort. Cancer Med. 2019;8(4):1406‐1415. 30802002 10.1002/cam4.2003 PMC6488109

[13] Scher HI, Morris MJ, Stadler WM, et al. Prostate cancer clinical trials working group. Trial design and objectives for castration‐resistant prostate cancer: updated recommendations from the prostate cancer clinical trials working group 3. J Clin Oncol. 2016;34(12):1402‐1418. 26903579 10.1200/JCO.2015.64.2702 PMC4872347

[14] Gibbons JA, De Vries M, Krauwinkel W, et al. Pharmacokinetic drug interaction studies with enzalutamide. Clin Pharmacokinet. 2015;54:1057‐1069. 25929560 10.1007/s40262-015-0283-1 PMC4580724

[15] Hendrikx JJ, Hillebrand MJ, Thijssen B, et al. A sensitive combined assay for the quantification of paclitaxel, docetaxel and ritonavir in human plasma using liquid chromatography coupled with tandem mass spectrometry. J Chromatogr B Analyt Technol Biomed Life Sci. 2011;879(28):2984‐2990. 10.1016/j.jchromb.2011.08.034 21920826

[16] RC Team . R: A Language and Environment for Statistical Computing. Vienna, Austria: R Foundation for Statistical Computing; 2009.

[17] Franke RM, Carducci MA, Rudek MA, Baker SD, Sparreboom A. Castration‐dependent pharmacokinetics of docetaxel in patients with prostate cancer. J Clin Oncol. 2010;28(30):4562‐4567. 20855838 10.1200/JCO.2010.30.7025 PMC2974340

[18] Kenmotsu H, Tanigawara Y. Pharmacokinetics, dynamics and toxicity of docetaxel: why the Japanese dose differs from the western dose. Cancer Sci. 2015;106(5):497‐504. 25728850 10.1111/cas.12647 PMC4452149

[19] Halabi S, Dutta S, Tangen CM, et al. Overall survival of black and white men with metastatic castration‐resistant prostate cancer treated with docetaxel. J Clin Oncol. 2019;37(5):403‐410. 30576268 10.1200/JCO.18.01279 PMC6804881

[20] Bruno R, Hille D, Riva A, et al. Population pharmacokinetics/pharmacodynamics of docetaxel in phase II studies in patients with cancer. J Clin Oncol. 1998;16(1):187‐196. 9440742 10.1200/JCO.1998.16.1.187

[21] Baker SD, Li J, ten Tije AJ, et al. Relationship of systemic exposure to unbound docetaxel and neutropenia. Clin Pharmacol Ther. 2005;77(1):43‐53. 15637530 10.1016/j.clpt.2004.09.005

[22] Yu H, Janssen JM, Sawicki E, et al. A population pharmacokinetic model of oral docetaxel coadministered with ritonavir to support early clinical development. J Clin Pharmacol. 2019;28(30):4562‐4567. 10.1002/jcph.1532 31595980

