# Supplementary material for: ModraDoc006, an oral docetaxel formulation in combination with ritonavir (ModraDoc006/r), in metastatic castration‐resistant prostate cancer patients: A phase Ib study
Source: Cancer Rep (Hoboken). 2021 Mar 12;4(4):e1367. doi: 10.1002/cnr2.1367 (PMC8388171; doi:10.1002/cnr2.1367)
Supplement: Supplementary file 1 — Data S1. Supporting Information. [file CNR2-4-e1367-s001.docx]

**Supplementary data**

*Table S1. Pharmacokinetic results of docetaxel and ritonavir*

| **Para-meter** | **Pt** | **Cohort 1**  **M:30-20mg**  **R: 100-100mg**  **(n=5)** | | | | | **Cohort 2**  **M: 30-20mg**  **R: 200-200mg**  **(n=6)** | | | | **Cohort 3A**  **M: 30-20mg**  **R: 200-100mg**  **(n=6)** | | | | **Cohort 3B**  **M: 20-20mg**  **R: 200-100mg**  **(n=3)** | | | |
| --- | --- | --- | --- | --- | --- | --- | --- | --- | --- | --- | --- | --- | --- | --- | --- | --- | --- | --- |
|  |  | **Docetaxel** | | **Ritonavir** | | | **Docetaxel** | | **Ritonavir** | | **Docetaxel** | | **Ritonavir** | | **Docetaxel** | | **Ritonavir** | |
| Cycle |  | 1 | 2 | 1 | | 2 | 1 | 2/5* | 1 | 2/5* | 1 | 2 | 1 | 2 | 1 | 2 | 1 | 2 |
| AUC_0-inf_ | 1 | 485* | 611* | 5721* | | 7402* | 289 | 332 | 18135 | 14934 | 679 | 877 | 21010 | 23222 | 435 | 666 | 14432 | 21242 |
|  | 2 | 283 | 256 | 14918 | | 12503 | 3418 | 3131 | 85263 | 98185 | 1325 | 1764 | 22847 | 27540 | 728 | 578 | 23054 | 15971 |
|  | 3 | 108 | 187 | 7713 | | 9972 | 1468 | 1458 | 41959 | 45979 | 1029 | 1391 | 41171 | 78579 | 416 | 525 | 8941 | 12920 |
|  | 4 | 565* | 862* | 8773** | | 9550* | 1644* | 2239* | 52893* | 44609* | 1116 | 1158 | 34188 | 35659 |  | |  | |
|  | 5 | 553 | 702 | 11042 | | 11547 | 1629 | 2611 | 50113 | 64784 | 2148 | 2478 | 43635 | 45072 |  |  |  |  |
|  | 6 |  | |  | | | 873 | 1157 | 35037 | 44734 | 1756 | 2484 | 26354 | 27372 |  |  |  |  |
|  | μ | 399  CV% 49.6  ( | 524  CV%  55.5 | 9514  CV%  38.0 | | 10195  CV%  19.3 | 1554  CV%  67.9 | 1821  CV%  56.5 | 47233  CV%  47.4 | 52204  CV%  52.9 | 1342  CV%  39.6 | 1692  CV%  40.0 | 31534  CV%  30.4 | 39574  CV%  52.1 | 526  CV%  33.2 | 590  CV%  12.1 | 15476  CV%  46.0 | 16711  CV%  25.2 |
| C_max_ | 1 | 38.6* | 47.4* | 873* | | 847* | 39.9 | 37.8 | 1930 | 1830 | 226 | 84.3 | 1750 | 2430 | 63.3 | 78.6 | 2130 | 4210 |
|  | 2 | 24.5 | 14.7 | 1050 | | 500 | 263 | 204 | 8380 | 9720 | 148 | 155 | 2740 | 2830 | 53 | 43.7 | 3400 | 1990 |
|  | 3 | 6.21 | 19.9 | 1010 | | 2270 | 134 | 156 | 4450 | 5260 | 97 | 128 | 4620 | 5710 | 21.98 | 33.3 | 467 | 1080 |
|  | 4 | 41.8* | 94.6* | 507* | | 807* | 159* | 170* | 6470* | 4090* | 130 | 150 | 6620 | 5650 |  | |  | |
|  | 5 | 54.1 | 54.1 | 733 | | 1320 | 205 | 230 | 11000 | 9890 | 214 | 267 | 6070 | 5290 |  |  |  |  |
|  | 6 |  | |  | | | 74.8 | 104 | 2650 | 3880 | 170 | 235 | 4020 | 3170 |  |  |  |  |
|  | μ | 33.0  CV% 55.5 | 46.1  CV% 69.3 | 835  CV% 26.5 | 1149  CV% 60.2 | | 146  CV%  56.3 | 150.3  CV%  46.5 | 5813  CV%  60.0 | 5578  CV%  57.3 | 164  CV%  30.2 | 170  CV%  40.2 | 4303  CV%  43.6 | 4180  CV% 36.5 | 46.1  CV%  46.8 | 51.9  CV%  45.7 | 1999  CV%  73.6 | 2427  CV%  66.3 |

* patients using enzalutamide <28 days prior to initiation of ModraDoc006/r. PK sampling occurred at cycle 1 and 5 (instead of 1 and 2) in the patient in cohort 2

Abbreviations: M = ModraDoc006, R = ritonavir, n = number of patients, Pt = patient number, AUC_0-inf_  = area under the plasma concentration versus time curve from 0 to infinity, in ng/ml*h, C_max_ = maximum plasma concentration in ng/ml, μ= mean, CV% = coefficient of variation (percentage)

*Figure S1A. Plasma concentration versus time curves of the patients in cohort 3A*


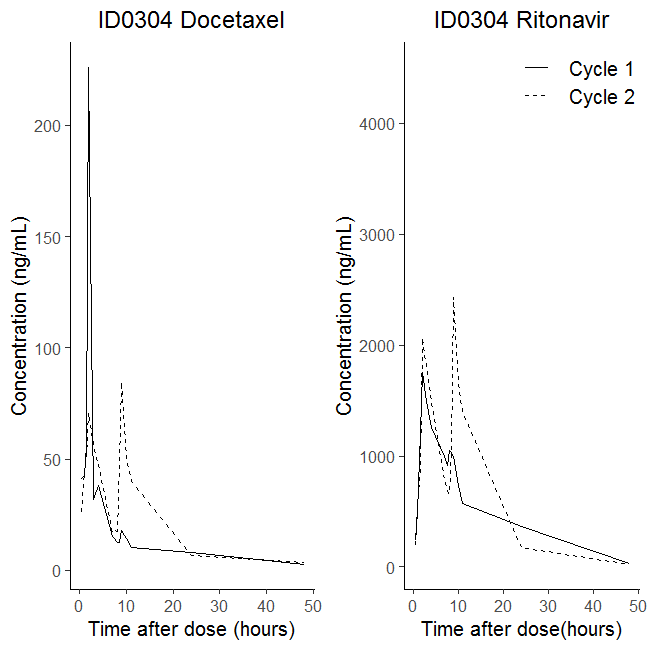

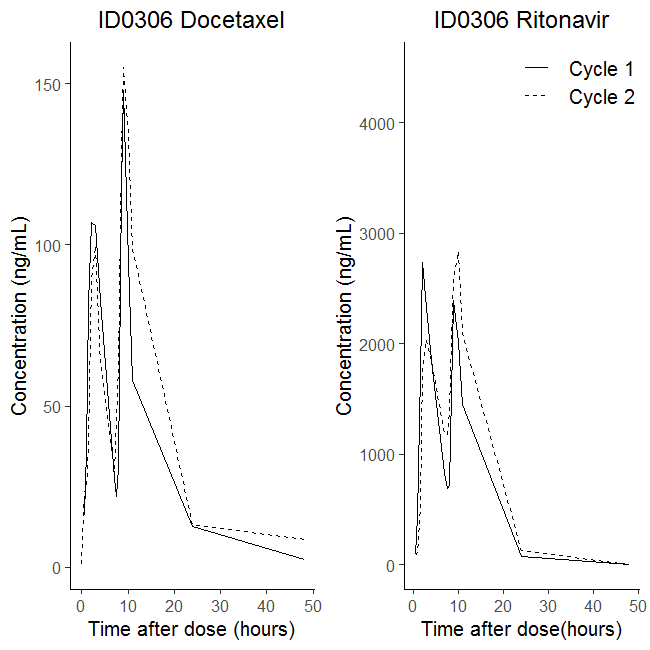

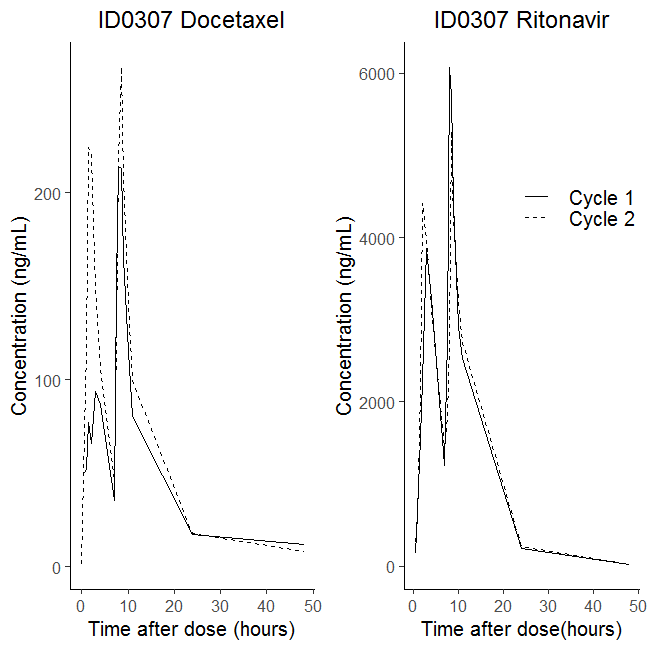


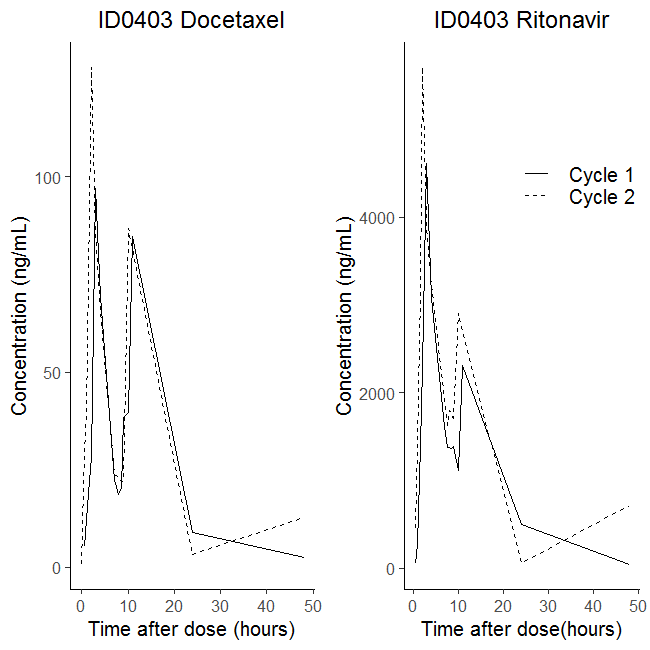

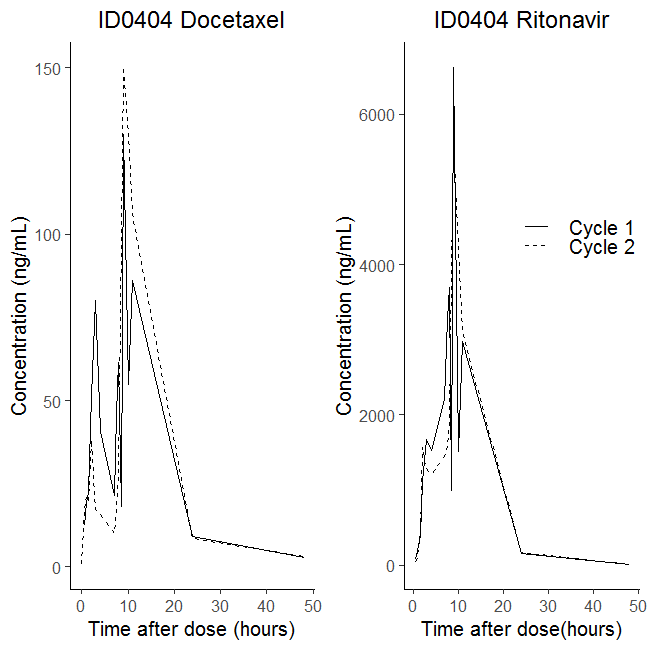

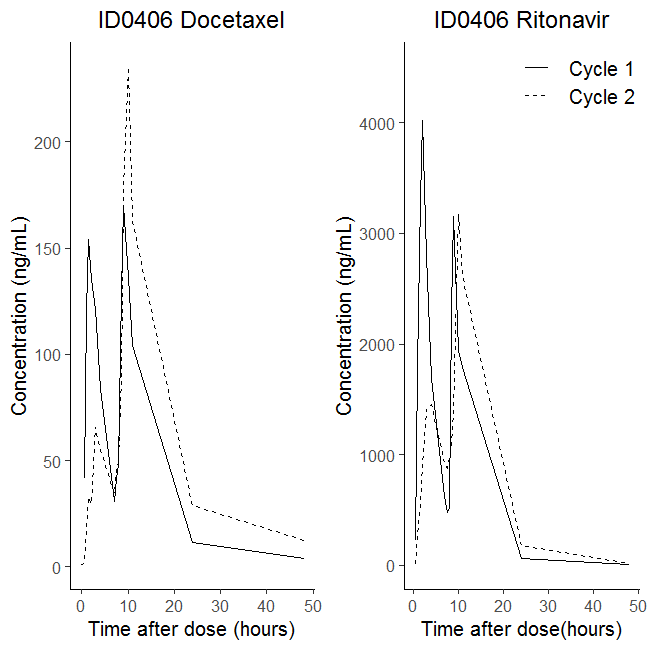


*Figure S1B. Plasma concentration versus time curves of the patients in cohort 3B*

*
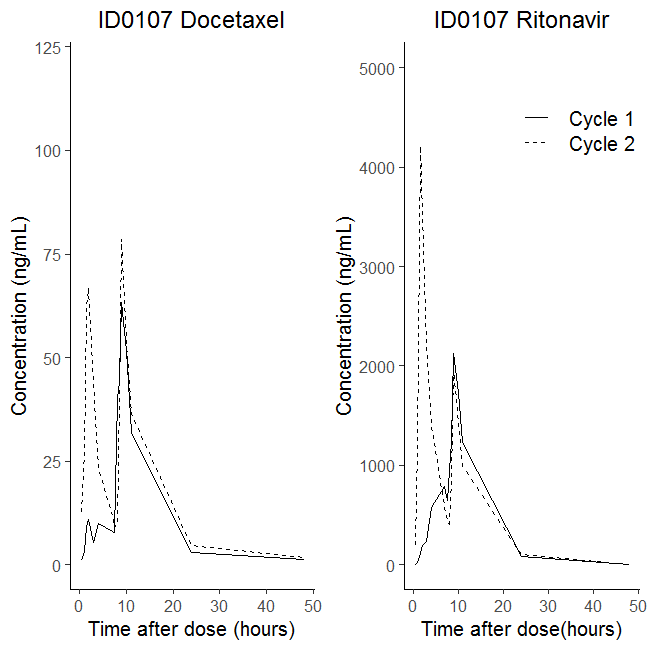

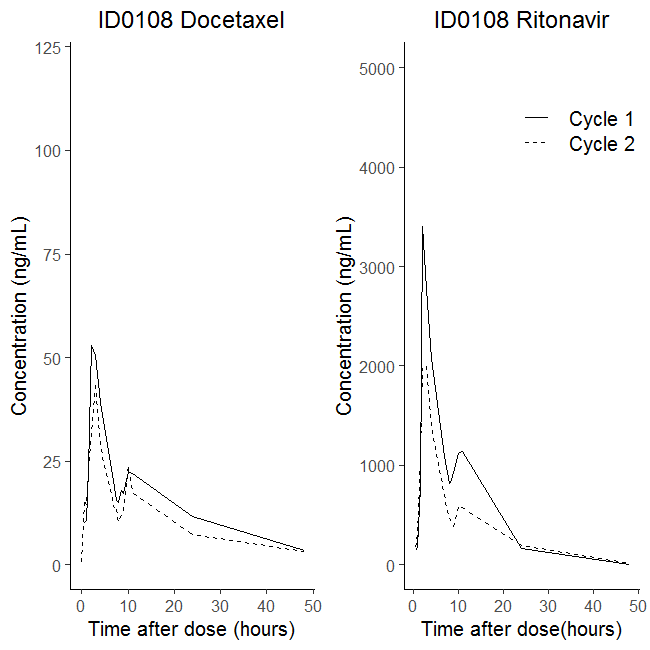

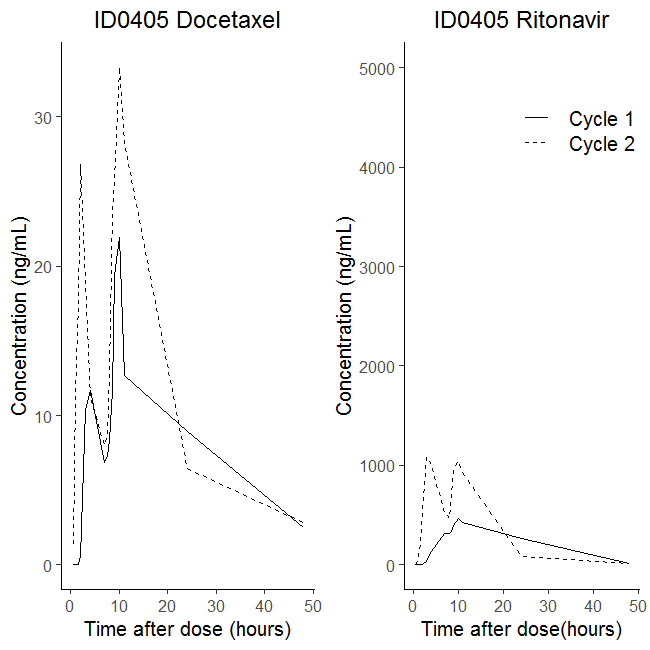
*
